# Cultivars identification of oat (*Avena sativa* L.) seed *via* multispectral imaging analysis

**DOI:** 10.3389/fpls.2023.1113535

**Published:** 2023-02-07

**Authors:** Xiuzhen Fu, Mengjie Bai, Yawen Xu, Tao Wang, Zhenning Hui, Xiaowen Hu

**Affiliations:** ^1^ State Key Laboratory of Herbage Improvement and Grassland Agro-ecosystems, Lanzhou University, Lanzhou, China; ^2^ Key Laboratory of Grassland Livestock Industry Innovation, Ministry of Agriculture and Rural Affairs, Lanzhou University, Lanzhou, China; ^3^ Engineering Research Center of Grassland Industry, Ministry of Education, Lanzhou University, Lanzhou, China; ^4^ College of Pastoral Agriculture Science and Technology, Lanzhou University, Lanzhou, China

**Keywords:** oat, seed feature, nondestructive identification, linear discriminant analysis, support vector machine

## Abstract

Cultivar identification plays an important role in ensuring the quality of oat production and the interests of producers. However, the traditional methods for discrimination of oat cultivars are generally destructive, time-consuming and complex. In this study, the feasibility of a rapid and nondestructive determination of cultivars of oat seeds was examined by using multispectral imaging combined with multivariate analysis. The principal component analysis (PCA), linear discrimination analysis (LDA) and support vector machines (SVM) were applied to classify seeds of 16 oat cultivars according to their morphological features, spectral traits or a combination thereof. The results demonstrate that clear differences among cultivars of oat seeds could be easily visualized using the multispectral imaging technique and an excellent discrimination could be achieved by combining data of the morphological and spectral features. The average classification accuracy of the testing sets was 89.69% for LDA, and 92.71% for SVM model. Therefore, the potential of a new method for rapid and nondestructive identification of oat cultivars was provided by multispectral imaging combined with multivariate analysis.

## 1 Introduction

Oat (*Avena sativa* L.), an annual grass species, is widely cultivated worldwide as one of the most important traditional human food as well as a healthy source of proteins, unsaturated fatty acids, vitamins, phenolics and significant quantities of dietary fibre, contributing to the beneficial health effects (Marshall et al., 2013; Singh et al., 2013; Menon et al., 2016). Meanwhile, oat is an important forage in cool semiarid regions (Carr et al., 2004). It also can be used as silage, pasture or hay and plays a vital role in sustainable agriculture related to animal production by providing forage in winter when warm-season grass production is limited by freezing temperatures (Maloney et al., 1999; Kim et al., 2017). It is well-known that with the continuous application of new breeding techniques, more and more cultivars oat available for cultivation are produced worldwide. Different oat cultivars show diverse adaptability and growth potential for their different genetic basis (Wang et al., 2019). For example, significant differences were found in seed yield in 196 native Turkish oats cultivars in 2-year field experiments (Dumlupinar et al., 2011). Likewise, the genotype (cultivar/strain) effect on the oat grain yield was the greatest, accounting for 61% of yield variation (Tobiasz-Salach et al, 2019). In addition, different cultivars showed different tolerance under salt stress (Zhang et al., 2022), heat stress (Rauf et al., 2020) and drought stress (Benlioglu and Ozkan, 2021). Studies have also shown that oat cultivars have a significant effect on the chemical component of oat (Silva et al., 2012; Tong et al., 2014). According to the purpose of cultivation and the different growth period of cultivars, selecting proper cultivars to cultivate in different places is an important means to ensure the quality of oat production. Therefore, effective cultivar identification to sort and ensure the purity of oat seeds is not only conducive to the healthy development of the seed industry to protect the breeding of new cultivars, but also conducive to the healthy development of agriculture to ensure the interests of producers.

Generally, cultivar identification is carried out on the basis of specific traits specified in official protocols during the registration process, such as seed shape, size, color and field seedling growth characteristics (Grillo et al., 2011). However, this identification process requires experienced and professional technicians, and the whole process is expensive and time-consuming. The high number of new cultivars and high similarity do not allow classified only based on phenotype (Dvořáček et al., 2003). Due to the continuous development and application of molecular marker technology in cultivar identification, molecular detection has been considered as an effective identification method with high precision (Saccomanno et al., 2020). RFLP (restriction fragment length polymorphism), RAPD (randomly amplified polymorphic DNA), SSR (simple sequence repeat) and other molecular markers have been successfully applied in oat cultivars identification (O’Donoughue et al., 1994; Paczos-Grzeda, 2004; Wight et al., 2010; Pipan et al., 2021). However, the lack of validated markers is one of the constraints of using molecular method (Sakiyama et al., 2014), and it is generally conducted destructive. Therefore, the potential of using non-destructive methods such as imaging techniques, NIR spectroscopy or precise remote sensors to overcome the limitations of traditional methods is gaining increasing attention.

The use of image analysis technology and measurement of the geometric shape of single seed can provide an alternative to manual inspection of seed for characteristic properties, thus the information could be visually obtained much faster (Geetha et al., 2011; Grillo et al., 2011; Sumathi and Balamurugan, 2013). Near-infrared spectroscopy (NIRS), as a sensing technology, is for the determination of chemical composition, both on the seed surface and inside the seeds (Boelt et al., 2018), providing potential for cultivar identification. Multispectral imaging, as an emerging technology, combines imaging and spectroscopy techniques, which can not only provide spatial information of seeds such as area, perimeter, length, shape, color, etc., but also provide detailed information about chemical composition, structure and other internal characteristics, determining various traits of seeds simultaneously. The merits of non-destructive, easy and no sample pre-treatment make it widely applied to seed quality testing, such as identifying rice seeds (Liu et al., 2016b), classifying different tomato seed cultivars (Shrestha et al., 2016), discriminating alfalfa cultivars seed (Yang et al., 2020; Jia et al., 2022), classifying *Jatropha curcas* seed health (Barboza da Silva et al., 2021). Sumathi and Balamurugan applied machine vision technology to identify the morphology of 11 oat cultivars, and the observation was more accurate and objective than manual method (Sumathi and Balamurugan, 2013). However, there is no report on the application of multispectral imaging technique for rapid recognition for oat cultivars.

From the above, our study aimed to develop a non-destructive, rapid and high-throughput oat cultivars identification method based on multispectral imaging technology combined with multivariate analysis.

## 2 Material and methods

### 2.1 Seed samples

**Figure 1:**
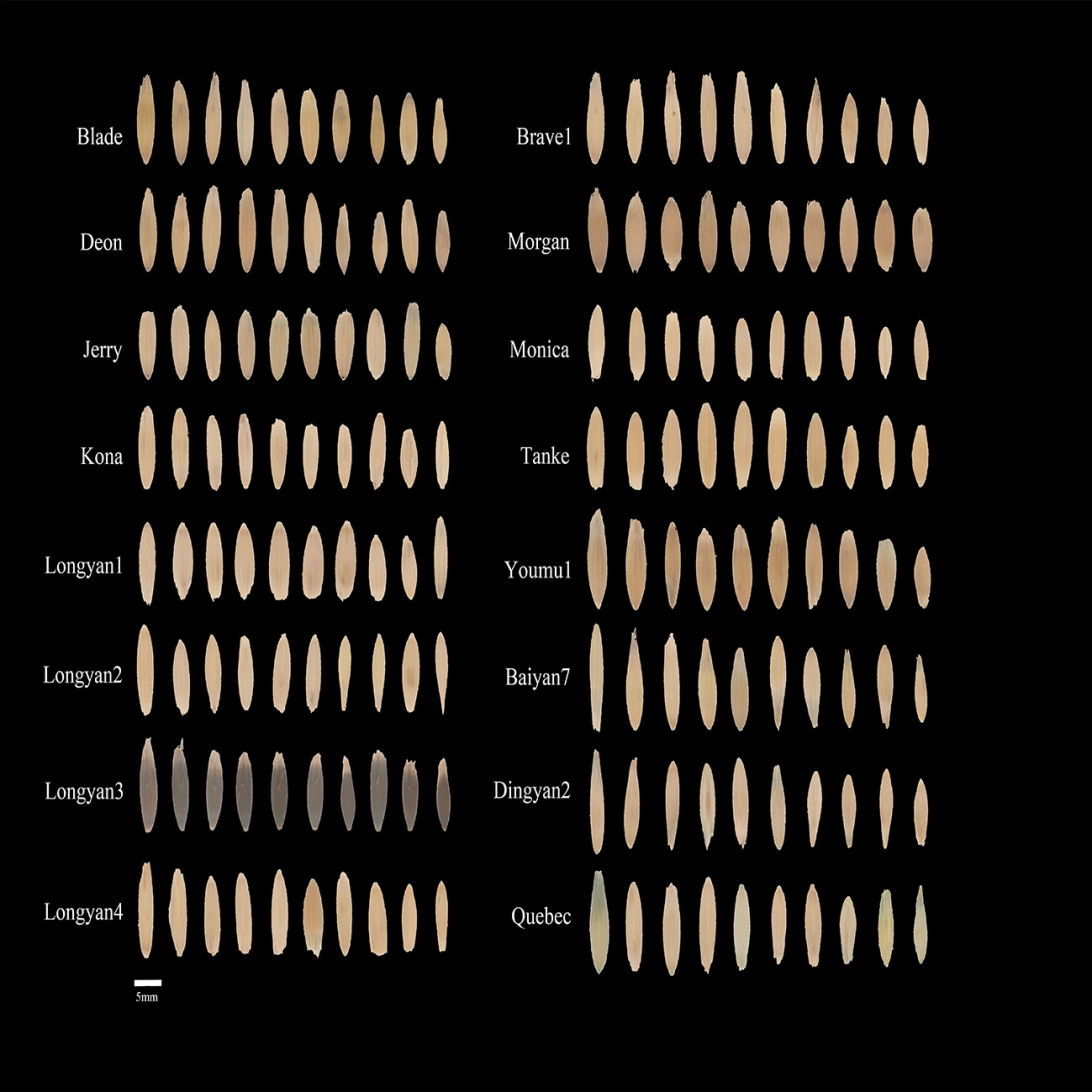
Seed image of 16 cultivars for *Avena sativa* L.

Sixteen oat cultivars were Blade, Deon, Jerry, Kona, Longyan1, Longyan2, Longyan3, Longyan4, Brave1, Morgan, Monica, Tanke, Youmu1, Baiyan7, Dingyan2 and Quebec. The seeds of Dingyan2, Baiyan7 and Quebec were provided by the Academy of Agricultural Science, Dingxi, Gansu Province; and the other cultivars were provided by Beijing Best Grass Industry Co., Ltd. Photo of the seeds is displayed in **Figure 1**.

For each cultivar, 200 seeds were used for classification experiments in January 2022. For each species, 140 seeds for each sample were randomly selected as training set and remaining 60 seeds were used for independent testing set.

### 2.2 Multispectral imaging system and seed imaging

Multispectral images were captured with a VideometerLab4 (Videometer, Hørsholm, Denmark) multispectral imaging system which is the same as the study by Hu et al. (2020). Samples were placed beneath a hollow integrating sphere, with a camera located in the top of the sphere. During image capture, the sphere closes over the sample stage to create optically closed conditions, allowing even lighting with minimal shadows and specular reflection. Samples were illuminated by 19 high power light emitting diodes (LEDs) at specific wavelengths: 365, 405, 430, 450, 470, 490, 515, 540, 570, 590, 630, 645, 660, 690, 780, 850, 880, 890, and 970 nm. The detailed information about VideometerLab4 correction have been described in their study.

Seeds were kept in water-proof bags in a storage room with average temperature of -18°C before imaging. During seed imaging, disease-free oat seeds were selected. For 200 seeds of each cultivar, 25 seeds (for 8 times) laid in a petri dish were used for image acquiring with the VideometerLab4 by controlling the ball falling and closing the top camera. The seeds were evenly arranged without contact to ensure that the extraction of the characteristic value of a single seed was not affected in the later stage.

### 2.3 Multispectral image analysis

The main objects appearing in the multispectral image are the 25 oat seeds in addition to some other objects, such as the petri dish and its surrounding background that should be removed from the image before extracting morphological spectral information of the individual seed. Thus, use a normalized canonical discriminant analysis (nCDA) in the VideometerLab software version 3.12 to segment the acquired image preliminarily and produce an image mask where only the oat seeds were isolated from non-seed pixels. The nCDA is a dimension-reduction method that maximized variation between the classes and reduced variation within a class (Olesen et al., 2015).

Then, attributes of the seeds such as morphological traits and main spectral features of all individual seeds were extracted from the image analysis and processed. The morphological traits included area, length, width, perimeter, width/length ratio, compactness circle, compactness ellipse, BetaShape_a, BetaShape_b, vertical skewness, CIELab L*, CIELab a*, CIELab b*, saturation and hue. The area, length, width and perimeter are binary features of the seeds, which are used to show seed size. Width/length ratio is the ratio of width to length of the sample. Compactness circle and compactness ellipse represent respectively the ratio of the measured object area to the area of a circle of the same length and the ratio to the area of an ellipse of the same length and width. BetaShape_a and BetaShape_b can be understood as endpoint parameters at both ends of the image. Vertical skewness refers to the skewness around the horizontal central axis. As shape features, all of them are described the shape and outlines characteristics of measured object. The saturation is defined as the brilliant of the sample color, and hue, as the biggest feature of color, is the appearance of the color. The L*a*b* is an international standard for color measurement proposed by Commission International d’Eclairage (CIE) in 1976. L* is the luminance component, and a* represents from green to red color and b* from blue to yellow are the two chromatic components (Papadakis et al., 2000). The extracted seed spectral signatures represent the mean intensity of the reflected light for each single wavelength calculated from all seed pixels in the image.

### 2.4 Multivariate data analysis

Multivariate analysis, including principal component analysis (PCA), linear discrimination analysis (LDA) and support vector machines (SVM), was conducted using *FactoMineR, MASS*, and *e1071* packages in R 2.4.1 respectively, to classify and distinguish the oat cultivar seed (Hu et al., 2020).

#### 2.4.1 PCA

Principal component analysis (PCA), the most commonly used unsupervised exploratory multivariate data analysis technique, was carried out to identify the patterns hidden in the morphological features and spectral data of all extracted seeds (ElMasry et al., 2019). PCA is often used to reduce the dimension of the dataset and resolve most of the information in the original variables with fewer variables, thus transforming multiple variables into few comprehensive indicators, namely principal components, without losing important information. The complex factors are grouped into several PCs by PCA, and unknown samples to be classified. In this work, PCA was employed to investigate the classification of the oat seeds.

#### 2.4.2 LDA

Linear discriminant analysis (LDA), as a classical and supervised machine learning algorithm which minimizes the with-class distance and maximizes the between-class distance simultaneously, thus achieving maximum discrimination, mainly used for prediction and sample recognition and classification (ElMasry et al., 2019; Yu et al., 2021). In this study, seeds of each cultivar were randomly divided into 140 seeds (70% of total samples) as training and 60 seeds (remaining 30%) as testing sets. The models for LDA classification were built using the training packages.

For each discriminant model, the recognition levels of samples in the testing set were the proportion of the number of seeds correctly identified to the total number of seeds in the testing set, which are calculated using the following equation.


(eq.1) $$\text{Accuracy}\left(\%\right)=\frac{\text{Correctly classified seeds}}{\text{Total number of seeds}}\times 100$$

#### 2.4.3 SVM

Support vector machine (SVM), as a supervised machine learning model, has been widely used in classification and nonlinear function estimation (Wang and Hu, 2005). As for multivariate function estimation or non-linear classiffcation tasks, SVM performed effectively. Different from other analysis methods, SVM can use fewer training variables or samples in high-dimensional characteristic space. It has proven to be powerful in NIR spectra classification (Devos et al., 2009).

The “Accuracy” of discrimination was determined according to Equation (1).

## 3 Results

### 3.1 Seed morphologic features

**Figure 2:**
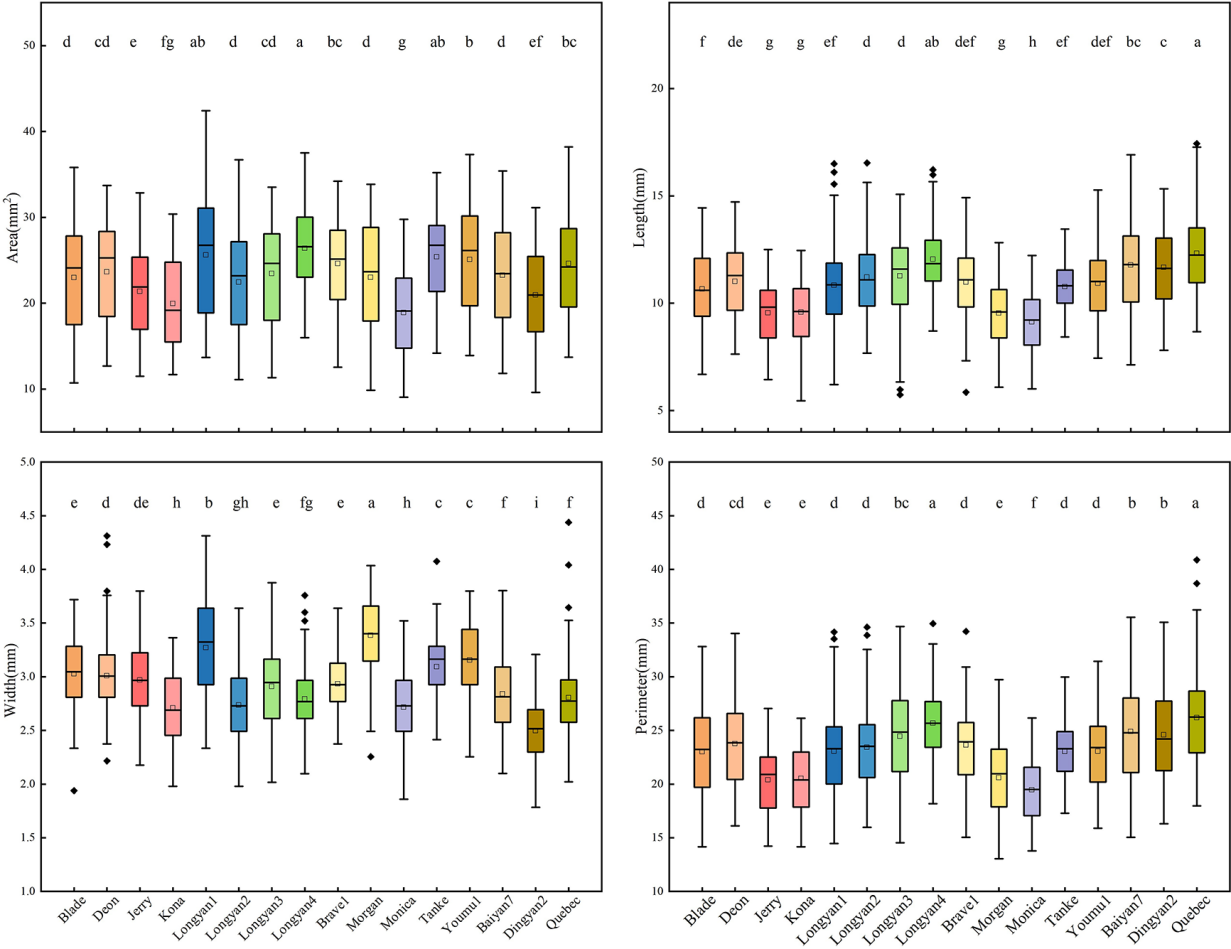
Binary features of 16 *Avena sativa* L. cultivars. Different lowercase letters represent significant differences between cultivars with the same feature (P<0.05).

For binary features, there were significant differences among 16 cultivars. The seeds of Longyan4 in area were the largest, and of Monica were the smallest. The length and perimeter of Monica were significantly lower than those of the other cultivars (**Figure 2**).

**Figure 3:**
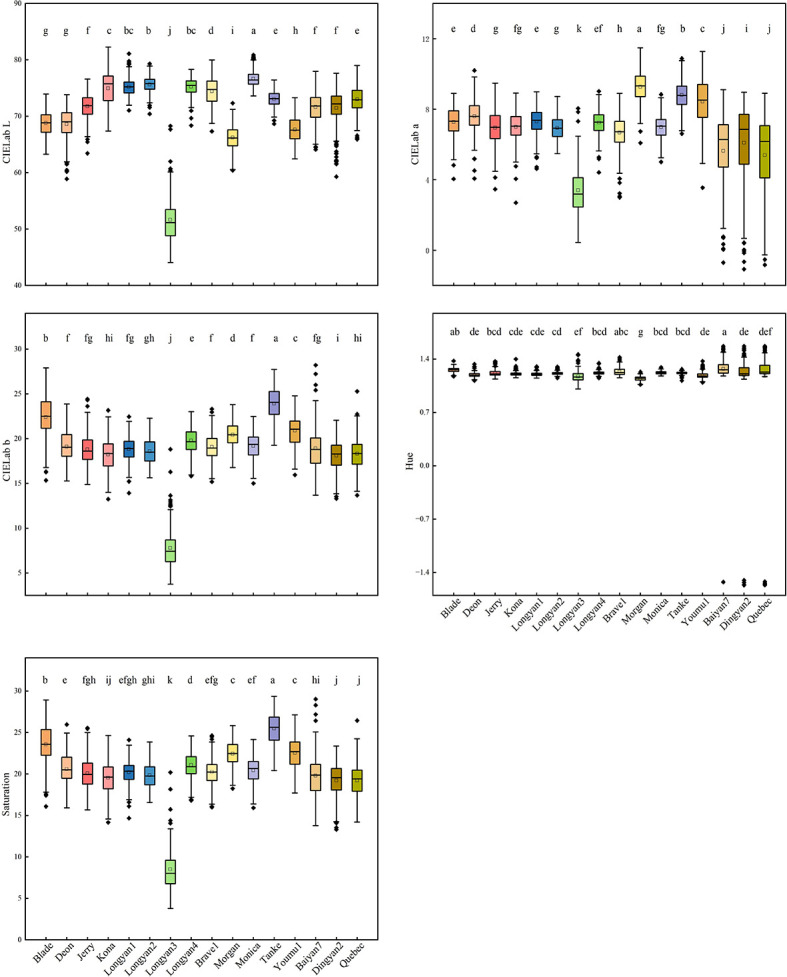
CIELab L*a*b*, saturation and hue features of 16 *Avena sativa* L. cultivars. Different lowercase letters represent significant differences between cultivars with the same feature (P<0.05).

For color features, 16 cultivars were different. The mean values of CIELab L* of Monica, CIELab a* of Morgan, CIELab b* and saturation of Tanke were the highest, the hue values of Baiyan7, Brave1 and Blade were significantly higher than the other cultivars. Longyan3, whose mean values of CIELab L*、CIELab a*、CIELab b* and saturation were the lowest, and the hue was only higher than that of Morgan (**Figure 3**).

**Figure 4:**
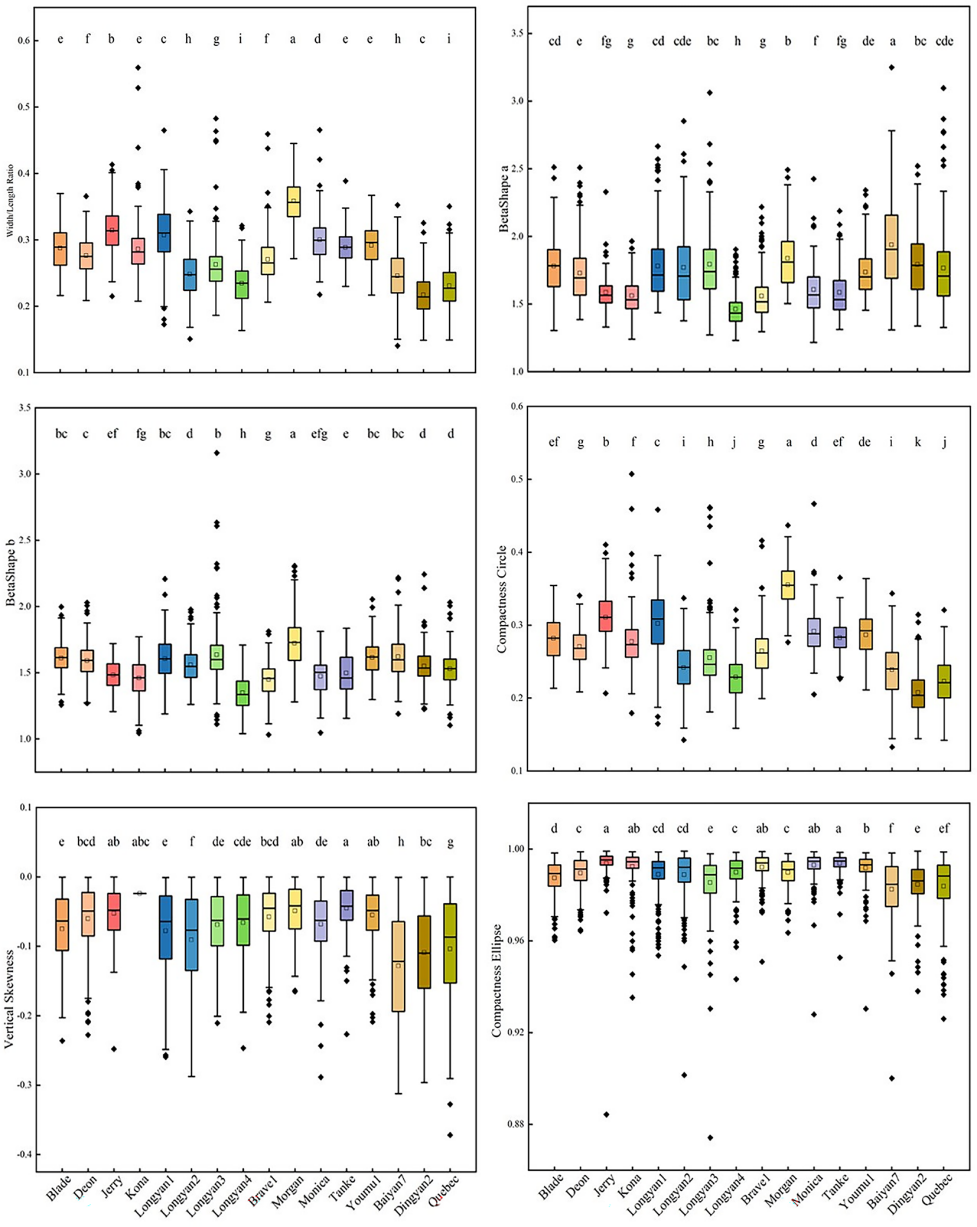
Shape features of 16 *Avena sativa* L. cultivars. Note: Different lowercase letters represent significant differences between cultivars with the same feature (P<0.05).

For shape features, significant differences were found among 16 cultivars. The width/length ratio, BetaShape_b and compactness circle of Morgan had the largest values. Baiyan7 had the highest value of BetaShape_a, but the lowest values of compactness circle, compactness ellipse and vertical skewness (**Figure 4**).

### 3.2 Spectroscopic analysis

**Figure 5:**
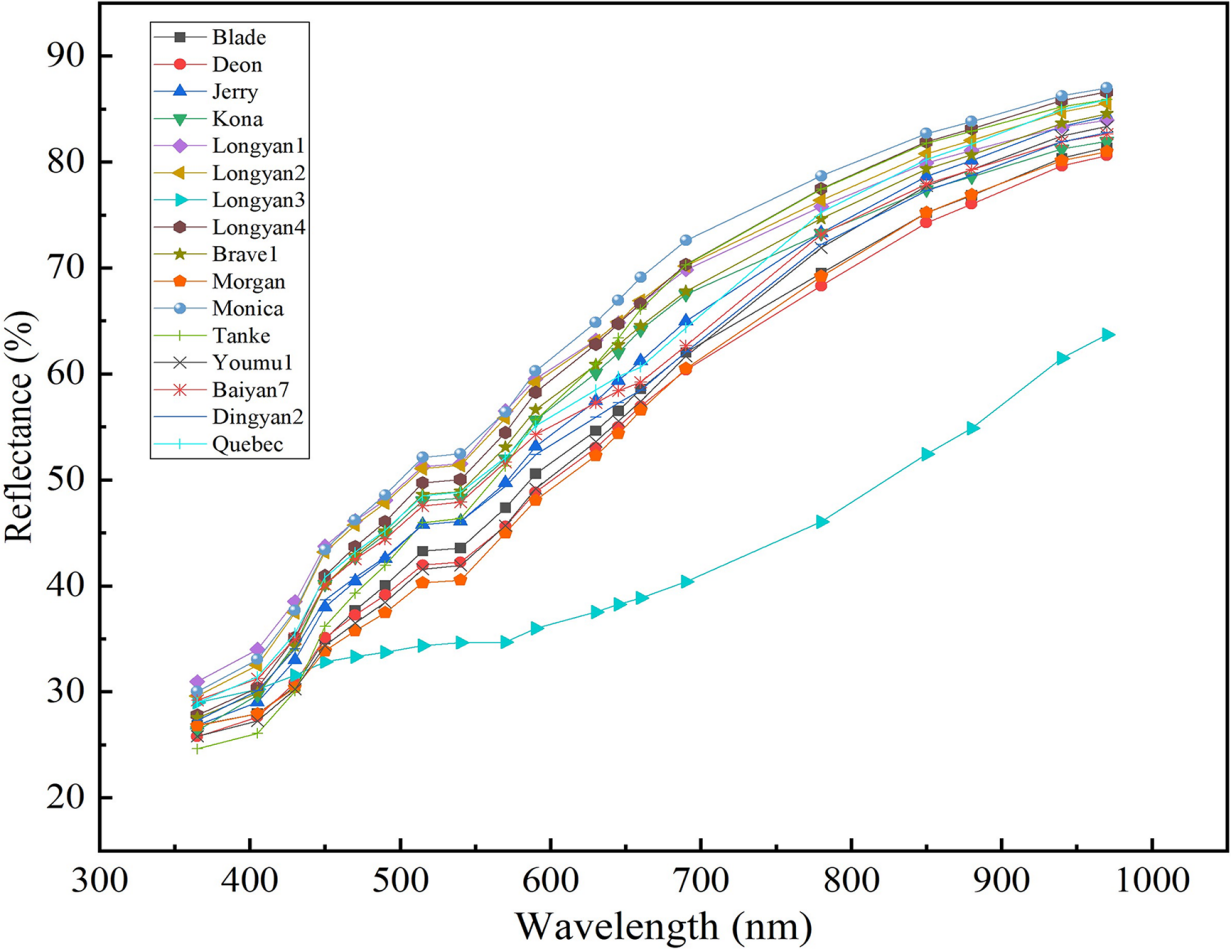
Mean light reflectance (%) of 19 wavelengths (nm) in 16 *Avena sativa* L. cultivars.

The spectral reflectance curves of oat seeds of different cultivars were smooth and had the similar trend across the whole wavelength region, the longer the wavelength, the higher the average reflectance (**Figure 5**). The reflectivity of different cultivars differed significantly at the same wavelength (**Supplementary Table 1**). For example, the average reflectivity of Monica was significantly higher than that of the other cultivars in the range of 515-970nm; while that of Longyan3 is significantly lower in the spectral range from 450 to 970 nm than the other cultivars.

### 3.3 PCA analysis

**Figure 6:**
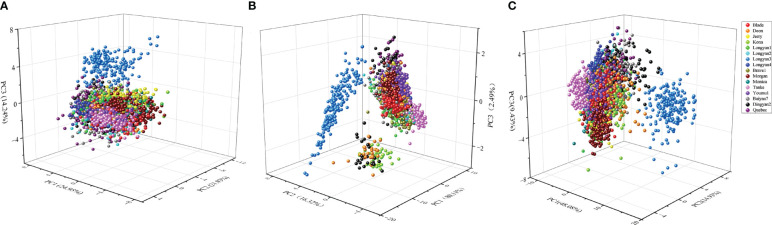
Three-dimensional plot of the first three principal components (PCs) for **(A)** morphological, **(B)** spectral and **(C)** morphological combined with spectral features dataset in 16 cultivars.

For morphological traits, the first three principal components could explain 61.12% of the variation among cultivars with 24.95, 21.93 and 14.24%, respectively (**Figure 6A**). For spectral features, the explained variance rates for the first three principal components were 80.11, 16.32 and 2.49%, respectively (**Figure 6B**). In addition, PCA results based on morphological characteristics and seed spectra showed that the first three principal components could explain 72.44% of the original variance among seeds with 48.08, 14.93 and 9.43% for PC1, PC2 and PC3, respectively (**Figure 6C**). However, either the score plot of PCA based on morphological, spectral data or the combination of the two failed to distinguish seeds of different cultivars into 16 groups.

### 3.4 Identification of cultivars by LDA model

**Figure 7:**
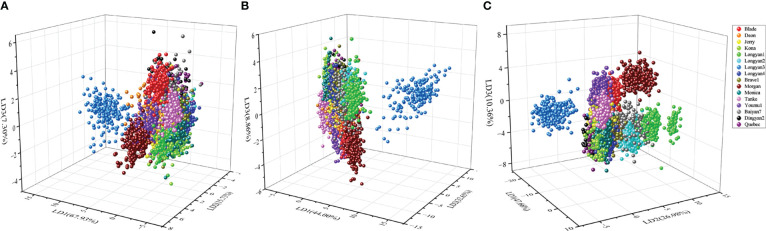
Scores plot of linear discrimination analysis (LDA) model for discrimination 16 cultivars seeds of *Avena sativa* L. based on **(A)** morphological, **(B)** spectral and **(C)** morphological combined with spectral features.

The LDA model based on morphological features data had an average classification accuracy of 61.16% and 60.63% for training and independent testing datasets, respectively (**Supplementary Table 2**; **Figure 7A** In contrast, the LDA model based on spectral data had a great improvement in the accuracy of cultivars discrimination, the overall discrimination accuracy was as high as 83.88% and 80.92% for the training and testing datasets, respectively (**Supplementary Table 3**; **Figure 7B** The LDA model combining morphological features and spectral data had the highest discrimination accuracy, with the value of 89.69% for testing datasets (**Table 1**; **Figure 7C**).

**Table 1:** Discrimination performance based on LDA with morphology and spectral features of 16 *Avena sativa* L. cultivars.

	Predict	Actual																Total (%)
		Blade	Deon	Jerry	Kona	Longyan1	Longyan2	Longyan3	Longyan4	Brave1	Morgan	Monica	Tanke	Youmu1	Baiyan7	Dingyan2	Quebec	
Testing (60)	Blade	53	0	2	0	0	0	0	0	0	0	0	0	0	2	0	0	
	Deon	4	57	3	0	0	0	0	0	2	0	0	0	0	0	1	3	
	Jerry	0	3	48	0	0	0	0	0	0	0	0	0	2	0	0	2	
	Kona	0	0	1	46	0	0	0	2	0	0	2	0	0	0	1	0	
	Longyan1	0	0	0	0	58	1	0	0	0	0	0	0	0	0	0	0	
	Longyan2	2	0	0	0	2	55	0	0	0	0	0	0	0	2	0	0	
	Longyan3	0	0	0	0	0	0	60	0	0	0	0	0	0	0	0	0	
	Longyan4	0	0	0	1	0	0	0	49	4	0	0	1	0	0	1	1	
	Brave1	1	0	1	0	0	2	0	6	51	0	0	0	0	0	1	4	
	Morgan	0	0	0	0	0	0	0	0	0	60	0	0	0	0	0	0	
	Monica	0	0	4	13	0	0	0	0	3	0	58	0	0	0	0	0	
	Tanke	0	0	0	0	0	0	0	0	0	0	0	59	0	0	0	0	
	Youmu1	0	0	1	0	0	0	0	0	0	0	0	0	56	0	0	0	
	Baiyan7	0	0	0	0	0	2	0	0	0	0	0	0	0	56	0	0	
	Dingyan2	0	0	0	0	0	0	0	1	0	0	0	0	0	0	49	4	
	Quebec	0	0	0	0	0	0	0	2	0	0	0	0	2	0	7	46	
	**Accuracy (%)**	88.33	95.00	80.00	76.67	96.67	91.67	100.00	81.67	85.00	100.00	96.67	98.33	93.33	93.33	81.67	76.66	89.69

However, the discriminant accuracy of different cultivars varied greatly. The discrimination accuracy of Longyan3 in the testing datasets reached 100%, while that of Brave1 was low as to 31.67%, when the model based on the morphology features data (**Supplementary Table 2**). Similarly, the discriminant accuracy of Longyan3 and Morgan was 100%, while the discrimination accuracy in both Kona and Quebec was less than 80% (**Table 1**).

### 3.5 Identification of cultivars by SVM model

The classification accuracy of the training and the testing datasets of SVM model based on the morphological feature data of the 16 cultivars was 66.83% and 66.25%, respectively (**Supplementary Table 4**). When the discrimination is based on the spectral data, the accuracy reached 88.79% and 88.65%, respectively (**Supplementary Table 5**). The overall discrimination accuracy of training and testing set based on the combination of morphological and spectral data was 95.49% and 92.71%, respectively (**Table 2**).

**Table 2:** Discrimination performance based on SVM with morphology and spectral features of 16 *Avena sativa* L. cultivars.

	Predict	Actual																Total (%)
		Blade	Deon	Jerry	Kona	Longyan1	Longyan2	Longyan3	Longyan4	Brave1	Morgan	Monica	Tanke	Youmu1	Baiyan7	Dingyan2	Quebec	
Testing (60)	Blade	56	1	0	0	0	0	0	0	0	0	0	0	0	2	0	0	
	Deon	4	52	1	0	1	0	0	0	1	1	0	0	0	2	1	2	
	Jerry	0	3	53	0	0	0	0	0	1	0	0	0	1	0	0	0	
	Kona	0	0	0	54	0	0	0	0	1	0	3	0	0	0	0	0	
	Longyan1	0	0	0	0	55	0	0	0	0	0	0	0	0	0	0	0	
	Longyan2	0	0	0	0	4	58	0	0	0	0	0	0	0	1	0	0	
	Longyan3	0	0	0	0	0	0	60	0	0	0	0	0	0	0	0	0	
	Longyan4	0	0	0	0	0	0	0	57	2	0	0	0	0	1	2	2	
	Brave1	0	1	2	0	0	2	0	2	53	0	0	0	0	0	0	1	
	Morgan	0	0	0	0	0	0	0	0	0	59	0	0	0	0	0	0	
	Monica	0	0	0	6	0	0	0	0	1	0	57	0	0	0	0	0	
	Tanke	0	0	1	0	0	0	0	0	0	0	0	60	0	0	0	0	
	Youmu1	0	1	1	0	0	0	0	0	0	0	0	0	59	0	0	0	
	Baiyan7	0	0	1	0	0	0	0	0	0	0	0	0	0	53	0	0	
	Dingyan2	0	2	1	0	0	0	0	0	0	0	0	0	0	1	54	5	
	Quebec	0	0	0	0	0	0	0	1	1	0	0	0	0	0	3	50	
	Accuracy (%)	93.33	86.67	88.33	90.00	91.67	96.67	100.00	95.00	88.33	98.33	95.00	100.00	98.33	88.33	90.00	83.33	92.71

Likewise, SVM model based on different types of data has different classification accuracy. For the model with morphology data, the accuracy of Longyan3 was 100%, while that of Brave1 was only 30% (**Supplementary Table 4**). When based on the spectral traits, there were 13 cultivars with more than 80% accuracy, while that of Jerry, Brave1 and Quebec was 76.67%, 78.33% and 66.67%, respectively (**Supplementary Table 5**). Using the combination of morphology features and spectral data, the accuracy of all cultivars reached more than 80%, the classification accuracy of Longyan3 and Tanke were 100%. Either using LDA or SVM model, Dingyan2 and Quebec have cross-error classification (**Table 2**).

## 4 Discussion

Although seeds of different oat cultivars differ a lot in morphological and spectral traits, any single seed trait failed to distinguish the seeds of different cultivars well due to a large variation within cultivars. PCA is an effective tool to classify oat cultivars into several groups by integrating several seed traits, while it failed to distinguish similar one, especially when trait variation is mainly from within cultivars rather than between cultivars. In contrast, the supervised learning algorithms, both LDA and SVM, using morphological, spectral features or their combination data all have a high accuracy in cultivar classification, in particularly, the overall accuracy of SVM model based on the combined data is higher than 90%.

### 4.1 Seed features variation

Whilst the morphological features clearly showed significant differences in binary, color and shape in terms of mean value among 16 cultivars, a large variation generally occurs within each cultivar, and thus neither of these traits are cultivar-specific, except for the dark seed coat color of Longyan3 which shows a distinct difference from the other 15 cultivars. This means cultivar classification based on single morphological traits is generally hard since variation within cultivars overrides the difference among cultivars. The result is similar with the study on seed identification of alfalfa cultivars (Yang et al., 2020). Moreover, as we expected, for physical eyes, morphological features similarity between cultivars was too high to be identified by any single feature.

Similar to morphological traits, the average spectral reflectance of different cultivars is different, which may relate to different physical and chemical components of seeds (Liu et al., 2016b). For example, the difference in visible light region may be concerned with the color of the seeds, and the difference in near-infrared region (NIR) may be related to variation in seed chemical composition among cultivars. Therefore, seed spectral trait provides abundant information for seed physical and chemical attributes, thus offers the potential in cultivar classification.

### 4.2 Unsupervised classification analysis

PCA, as an unsupervised exploratory pattern recognition technique, is commonly used to get an overview of the systematic variation in data, exploring the possibility of grouping the seeds (ElMasry et al., 2019; Yang et al., 2020). Previous studies have shown that conventional soybean, glyphosate-resistant soybean seeds and hybrid progeny were significantly separated from each other based on the distribution scatter plot of three-dimensional principal components (Liu et al., 2016a). In contrast, although PCA could distinguish the cultivars into several categories in our study, most of the cultivars cannot be successfully identified (**Figure 6**). It indicates that traits of the cultivars are differentiation, which were better than the use of a single trait to distinguish, and demonstrate that PCA has the potential for preliminary classification of multiple cultivars. However, the method failed to sperate similar cultivars. A possible reason is that PCA method aims to maximize the variance of cultivars rather than to maximize the ability to distinguish oat cultivars. Under the circumstances, if the mean of the between-group variables is very similar and the variance is large, the total variance will be mainly composed of within-group variance, not those between groups. Thus, PCA would not detect inter-group differences. In reality, both morphological and spectral features have very close mean values, and there is a large overlap and cross distribution among the seeds of oat cultivars. Besides, some important information was lost in the process of PCA dimensionality reduction, which is also one of the reasons why seeds failed to be discriminated well.

### 4.3 Supervised classification analysis

Supervised methods aim to minimize the distance within class and to maximize the distance between groups, thus models can show good discriminability among groups. As supervised discriminant models, LDA and SVM performed well in the process of identifying oat cultivars, especially when based on the combination of morphological and spectral features data of seeds for discrimination, the average accuracy of LDA and SVM models for oat cultivars identification was 89.69% and 92.71%, respectively. Yang et al. used LDA and SVM models to classify 12 alfalfa cultivars, and the classification accuracy of the testing set was 91.53% and 93.47%, respectively (Yang et al., 2020). However, it is also worth noting that there is a relatively low discrimination accuracy due to cross-error classification for some cultivars, such as Quebec and Dingyan2. In this case, a part of seeds of Quebec are misclassified as Dingyan2, and vice versa. The possible reason is that seed characteristics of different oat cultivars are affected by environmental factors as well as genetic factors, and the stability of the traits of different oat cultivars is diverse. For example, the protein content of wheat grain varied with the production region (Zhou et al., 2021). Oat seeds from the same cultivated area have similar growth environment and seed processing, which results in similar seed morphology and chemical composition. For instance, both Quebec and Dingyan2 seeds in our study are produced in Dingxi, which may lead to a similar maternal environmental effect for seed traits.

Although our study provided a rapid way to screen seeds of different oat cultivars using multispectral imaging technique and multivariate analysis, some limitations for present study is worthy noticed for its further application. Firstly, compared to the large number of oat cultivars across the world, 16 is a quite small number and thus limits the discrimination model application in a broader range. Thus, incorporating a huge sample size in future study will help to produce a universal, sharp and steady oat cultivar identification model *via* multispectral imaging techniques. Secondly, as we discussed above, seed traits could be affected by growth conditions as well as harvest and processing methods even for the same cultivar, thus, including various seed lots of each cultivar is necessary to account for genetic effect in the discrimination model. Thirdly, the VideometerLab4 multispectral imaging system used in our study including 19 specific wavelengths ranged from 365 to 970 nm only, the limited band range will inevitably lead to the loss of some information, thus limiting its application. In particularly for seed chemical composition, the spectral information is generally correlated with the reflectance at longer wavelengths. For example, the peak reflectance at 1,100nm may relate to the second overtone of C-H stretching, and so is the valley near 1,200nm (Zhang et al., 2016), the peak at 1,304.6 nm may be assigned to combination between the first with N-H in-plane bending vibrations (Zhang et al., 2017a), the valley near 1,450 nm is related to the first overtone of the tension and vibration inherent in O-H and N-H (Zhang et al., 2017b). These are related to the composition and content of chemical substances, such as fatty, protein and starch, resulting in different reflectivity. Thus, a wide range wavelength such as near infrared spectroscopy may help to improve the data quality and favor discrimination model building.

## 5 Conclusion

In brief, our study clearly shows that differences in seed features among oat cultivars did exist and could be visualized *via* the multispectral imaging technique. Both LDA and SVM, as supervised classification models, have high accuracy in classifying oat cultivars, especially when based on the combination of seed morphological and spectral features. It suggests that multispectral imaging together with multivariate analysis could be an efficient and non-destructive way for oat cultivar identification.

## Data availability statement

The original contributions presented in the study are included in the article/**Supplementary Material**. Further inquiries can be directed to the corresponding author.

## Author contributions

XH conceived and designed the experiment. XF performed the experiments and analyzed the data. MB, YX, TW and ZH contributed in the experiment. XF wrote and XH revised the paper. All authors contributed to the article and approved the submitted version.
