# Supplementary material for: Cultivars identification of oat (Avena sativa L.) seed via multispectral imaging analysis
Source: Front Plant Sci. 2023 Feb 7;14:1113535. doi: 10.3389/fpls.2023.1113535 (PMC9941542; doi:10.3389/fpls.2023.1113535)
Supplement: Supplementary file 1 [file Table_1.docx]

Supplementary Table 1. Mean reflectance of 16 *Avena sativa* L. cultivars at 19 wavelengths.

|  | Cultivar | | | | | | | | | | | | | | | |
| --- | --- | --- | --- | --- | --- | --- | --- | --- | --- | --- | --- | --- | --- | --- | --- | --- |
| Wavelength | Blade | Deon | Jerry | Kona | Longyan1 | Longyan2 | Longyan3 | Longyan4 | Brave1 | Morgan | Monica | Tanke | Youmu1 | Baiyan7 | Dingyan2 | Quebec |
| 365 | 26.83ef | 25.77g | 26.85ef | 26.34fg | 30.96a | 29.63bc | 28.98c | 27.82d | 27.53de | 26.80ef | 30.03b | 24.62h | 25.81g | 29.20c | 27.32de | 29.00c |
| 405 | 27.94f | 27.64f | 29.02e | 29.69de | 34.02a | 32.51b | 30.24d | 30.38d | 29.91d | 27.93f | 33.06b | 26.09g | 27.28f | 31.22c | 30.11d | 31.43c |
| 430 | 30.50h | 30.86gh | 33.02f | 34.56de | 38.52a | 37.47b | 31.57g | 35.15cd | 34.30de | 30.40h | 37.68b | 30.09h | 30.26h | 35.05cd | 34.05e | 35.56c |
| 450 | 34.95e | 35.11e | 38.00c | 40.16b | 43.75a | 43.18a | 32.85g | 40.96b | 40.08b | 33.85f | 43.41a | 36.18d | 34.38ef | 40.13b | 38.69c | 40.79b |
| 470 | 37.72f | 37.26fg | 40.44d | 42.70c | 46.14a | 45.74a | 33.31i | 43.71b | 42.86bc | 35.77h | 46.21a | 39.28e | 36.51gh | 42.50c | 40.81d | 43.18bc |
| 490 | 40.03e | 39.13f | 42.54d | 44.84c | 48.09a | 47.82a | 33.75h | 46.07b | 45.15c | 37.46g | 48.57a | 41.92d | 38.44f | 44.44c | 42.67d | 45.22c |
| 515 | 43.27g | 41.96h | 45.75f | 48.03de | 51.27b | 51.06b | 34.35j | 49.73c | 48.62d | 40.28i | 52.13a | 45.93f | 41.56h | 47.56e | 45.77f | 48.51d |
| 540 | 43.57g | 42.24h | 46.07f | 48.28de | 51.53b | 51.37b | 34.66j | 50.04c | 48.90d | 40.53i | 52.47a | 46.35f | 41.94h | 47.90e | 46.11f | 48.86d |
| 570 | 47.38f | 45.64g | 49.70e | 51.96d | 56.51a | 55.83a | 34.70h | 54.47b | 53.09c | 44.99g | 56.41a | 51.30d | 45.66g | 51.67d | 49.38e | 52.11d |
| 590 | 50.58h | 48.85ij | 53.16g | 55.64e | 59.55ab | 59.17b | 35.98k | 58.27c | 56.62d | 48.06j | 60.27a | 55.62e | 49.17i | 54.29f | 52.38g | 55.11e |
| 630 | 54.65h | 53.03ij | 57.44f | 60.07d | 63.19b | 63.12b | 37.51k | 62.80b | 60.86cd | 52.28i | 64.86a | 60.93c | 53.60i | 57.30f | 55.90g | 58.52e |
| 645 | 56.52g | 54.99hi | 59.36e | 62.05d | 64.82b | 64.90b | 38.25j | 64.73b | 62.74cd | 54.34i | 66.94a | 63.40c | 55.57h | 58.41f | 57.30g | 59.78e |
| 660 | 58.58e | 56.95f | 61.20d | 64.17c | 66.64b | 66.90b | 38.87g | 66.63b | 64.53c | 56.57f | 69.11a | 66.10b | 57.42f | 59.20e | 58.44e | 60.64d |
| 690 | 62.01ef | 60.38g | 64.97d | 67.51c | 69.82b | 70.20b | 40.39h | 70.32b | 67.79c | 60.49g | 72.58a | 70.29b | 61.68f | 62.66e | 62.09ef | 64.35d |
| 780 | 69.50h | 68.32i | 73.30f | 73.25f | 75.80cd | 76.39c | 46.03j | 77.48b | 74.65e | 69.19h | 78.68a | 77.43b | 71.88g | 73.18f | 72.23g | 75.24de |
| 850 | 75.18i | 74.27j | 78.63fg | 77.36h | 79.89de | 80.77c | 52.41k | 81.90ab | 79.32ef | 75.22i | 82.68a | 81.75b | 77.72h | 77.92gh | 77.28h | 80.23cd |
| 880 | 76.84h | 76.03i | 80.16f | 78.60g | 81.07de | 82.04c | 54.88j | 83.13ab | 80.68ef | 76.92h | 83.81a | 82.91b | 79.28g | 79.26g | 78.73g | 81.69cd |
| 940 | 80.34g | 79.63g | 83.37d | 81.24f | 83.31d | 84.70c | 61.47h | 85.79ab | 83.64d | 80.12g | 86.25a | 85.21bc | 82.45e | 81.91ef | 81.90ef | 84.95c |
| 970 | 81.35hi | 80.60i | 84.30d | 81.96gh | 83.95de | 85.52c | 63.70j | 86.60ab | 84.54d | 80.97i | 86.99a | 85.89bc | 83.36ef | 82.61fg | 82.76f | 85.90bc |

Note: Different lowercase letters represent significant differences in the mean reflectance between cultivars at the same wavelength (P<0.05).
