# Supplementary material for: Cultivars identification of oat (Avena sativa L.) seed via multispectral imaging analysis
Source: Front Plant Sci. 2023 Feb 7;14:1113535. doi: 10.3389/fpls.2023.1113535 (PMC9941542; doi:10.3389/fpls.2023.1113535)
Supplement: Supplementary file 2 [file Table_2.docx]

Supplementary Table 2. Discrimination performance based on LDA with morphology features of 16 *Avena sativa* L. cultivars.

|  | **Predict** | **Actual** | | | | | | | | | | | | | | | | **Total (%)** |
| --- | --- | --- | --- | --- | --- | --- | --- | --- | --- | --- | --- | --- | --- | --- | --- | --- | --- | --- |
|  |  | Blade | Deon | Jerry | Kona | Longyan1 | Longyan2 | Longyan3 | Longyan4 | Brave1 | Morgan | Monica | Tanke | Youmu1 | Baiyan7 | Dingyan2 | Quebec |  |
| Training | Blade | 119 | 5 | 4 | 1 | 0 | 0 | 0 | 1 | 0 | 0 | 0 | 0 | 11 | 12 | 4 | 2 |  |
| (140) | Deon | 4 | 72 | 10 | 10 | 0 | 0 | 2 | 0 | 7 | 0 | 0 | 0 | 12 | 6 | 5 | 5 |  |
|  | Jerry | 6 | 15 | 79 | 7 | 3 | 2 | 0 | 0 | 6 | 1 | 4 | 2 | 5 | 7 | 1 | 3 |  |
|  | Kona | 1 | 2 | 15 | 55 | 2 | 22 | 0 | 13 | 9 | 0 | 11 | 0 | 0 | 1 | 9 | 2 |  |
|  | Longyan1 | 0 | 1 | 5 | 4 | 99 | 9 | 0 | 2 | 3 | 2 | 8 | 2 | 0 | 2 | 1 | 1 |  |
|  | Longyan2 | 0 | 0 | 1 | 13 | 12 | 52 | 0 | 13 | 12 | 0 | 9 | 1 | 1 | 10 | 17 | 12 |  |
|  | Longyan3 | 0 | 0 | 0 | 0 | 0 | 0 | 138 | 0 | 0 | 0 | 0 | 0 | 0 | 0 | 0 | 0 |  |
|  | Longyan4 | 0 | 0 | 1 | 3 | 1 | 9 | 0 | 93 | 9 | 0 | 2 | 1 | 0 | 0 | 6 | 13 |  |
|  | Brave1 | 1 | 6 | 10 | 16 | 8 | 22 | 0 | 10 | 63 | 0 | 12 | 6 | 0 | 6 | 5 | 13 |  |
|  | Morgan | 0 | 1 | 0 | 1 | 1 | 0 | 0 | 0 | 0 | 126 | 0 | 0 | 12 | 0 | 0 | 0 |  |
|  | Monica | 0 | 0 | 2 | 23 | 7 | 7 | 0 | 2 | 14 | 0 | 94 | 0 | 0 | 1 | 1 | 1 |  |
|  | Tanke | 1 | 0 | 7 | 1 | 1 | 1 | 0 | 3 | 5 | 0 | 0 | 123 | 0 | 2 | 0 | 0 |  |
|  | Youmu1 | 3 | 28 | 4 | 0 | 0 | 1 | 0 | 0 | 2 | 11 | 0 | 5 | 89 | 3 | 2 | 1 |  |
|  | Baiyan7 | 3 | 5 | 1 | 0 | 4 | 4 | 0 | 0 | 2 | 0 | 0 | 0 | 7 | 65 | 8 | 25 |  |
|  | Dingyan2 | 1 | 4 | 0 | 5 | 0 | 3 | 0 | 3 | 6 | 0 | 0 | 0 | 3 | 9 | 62 | 21 |  |
|  | Quebec | 1 | 1 | 1 | 1 | 2 | 8 | 0 | 0 | 2 | 0 | 0 | 0 | 0 | 16 | 19 | 41 |  |
|  | **Accuracy (%)** | 85.00 | 51.43 | 56.43 | 39.29 | 70.71 | 37.14 | 98.57 | 66.43 | 45.00 | 90.00 | 67.14 | 87.86 | 63.57 | 46.43 | 44.29 | 29.29 | 61.16 |
| Testing | Blade | 46 | 1 | 1 | 1 | 0 | 0 | 0 | 0 | 0 | 0 | 0 | 0 | 6 | 7 | 3 | 0 |  |
| (60) | Deon | 3 | 37 | 4 | 1 | 0 | 0 | 0 | 0 | 7 | 0 | 0 | 0 | 6 | 3 | 0 | 4 |  |
|  | Jerry | 4 | 3 | 34 | 1 | 1 | 1 | 0 | 0 | 2 | 1 | 1 | 0 | 2 | 2 | 0 | 1 |  |
|  | Kona | 1 | 0 | 6 | 23 | 0 | 8 | 0 | 6 | 5 | 0 | 5 | 0 | 0 | 0 | 4 | 1 |  |
|  | Longyan1 | 0 | 1 | 1 | 0 | 43 | 6 | 0 | 1 | 1 | 1 | 4 | 1 | 0 | 3 | 0 | 1 |  |
|  | Longyan2 | 0 | 0 | 0 | 7 | 4 | 23 | 0 | 8 | 5 | 0 | 2 | 1 | 0 | 2 | 7 | 4 |  |
|  | Longyan3 | 0 | 0 | 0 | 0 | 4 | 0 | 60 | 0 | 0 | 0 | 0 | 0 | 0 | 0 | 0 | 0 |  |
|  | Longyan4 | 0 | 0 | 0 | 1 | 4 | 4 | 0 | 38 | 4 | 0 | 1 | 0 | 0 | 1 | 1 | 7 |  |
|  | Brave1 | 0 | 3 | 7 | 6 | 4 | 9 | 0 | 3 | 19 | 0 | 6 | 4 | 0 | 1 | 4 | 8 |  |
|  | Morgan | 0 | 1 | 0 | 0 | 4 | 0 | 0 | 0 | 0 | 52 | 0 | 0 | 1 | 0 | 0 | 0 |  |
|  | Monica | 0 | 0 | 0 | 20 | 4 | 2 | 0 | 0 | 8 | 0 | 41 | 0 | 0 | 0 | 0 | 0 |  |
|  | Tanke | 1 | 0 | 4 | 0 | 4 | 0 | 0 | 2 | 4 | 0 | 0 | 53 | 0 | 1 | 0 | 0 |  |
|  | Youmu1 | 3 | 6 | 2 | 0 | 4 | 0 | 0 | 0 | 0 | 6 | 0 | 1 | 40 | 0 | 0 | 1 |  |
|  | Baiyan7 | 2 | 3 | 1 | 0 | 4 | 2 | 0 | 0 | 1 | 0 | 0 | 0 | 4 | 31 | 4 | 10 |  |
|  | Dingyan2 | 0 | 3 | 0 | 0 | 4 | 1 | 0 | 2 | 3 | 0 | 0 | 0 | 1 | 4 | 26 | 7 |  |
|  | Quebec | 0 | 2 | 0 | 0 | 4 | 4 | 0 | 0 | 1 | 0 | 0 | 0 | 0 | 5 | 11 | 16 |  |
|  | **Accuracy (%)** | 76.67 | 61.67 | 56.67 | 38.33 | 71.67 | 38.33 | 100.00 | 63.33 | 31.67 | 86.67 | 68.33 | 88.33 | 66.67 | 51.67 | 43.33 | 26.67 | 60.63 |
