# Supplementary material for: Cultivars identification of oat (Avena sativa L.) seed via multispectral imaging analysis
Source: Front Plant Sci. 2023 Feb 7;14:1113535. doi: 10.3389/fpls.2023.1113535 (PMC9941542; doi:10.3389/fpls.2023.1113535)
Supplement: Supplementary file 3 [file Table_3.docx]

Supplementary Table 3. Discrimination performance based on LDA with spectral features of 16 *Avena sativa* L. cultivars.

|  | **Predict** | **Actual** | | | | | | | | | | | | | | | | **Total（%）** |
| --- | --- | --- | --- | --- | --- | --- | --- | --- | --- | --- | --- | --- | --- | --- | --- | --- | --- | --- |
|  |  | Blade | Deon | Jerry | Kona | Longyan1 | Longyan2 | Longyan3 | Longyan4 | Brave1 | Morgan | Monica | Tanke | Youmu1 | Baiyan7 | Dingyan2 | Quebec |  |
| Training | Blade | 117 | 0 | 0 | 0 | 0 | 0 | 0 | 1 | 1 | 0 | 0 | 0 | 0 | 7 | 0 | 1 |  |
| (140) | Deon | 10 | 125 | 14 | 0 | 0 | 0 | 2 | 0 | 6 | 0 | 0 | 0 | 9 | 3 | 6 | 5 |  |
|  | Jerry | 0 | 5 | 93 | 4 | 0 | 0 | 0 | 5 | 3 | 0 | 1 | 3 | 6 | 0 | 2 | 6 |  |
|  | Kona | 0 | 0 | 4 | 97 | 0 | 0 | 0 | 4 | 0 | 0 | 7 | 1 | 0 | 0 | 7 | 3 |  |
|  | Longyan1 | 0 | 0 | 0 | 0 | 126 | 4 | 0 | 0 | 0 | 4 | 0 | 0 | 0 | 0 | 0 | 0 |  |
|  | Longyan2 | 3 | 0 | 0 | 0 | 14 | 129 | 0 | 0 | 1 | 1 | 0 | 0 | 0 | 8 | 0 | 0 |  |
|  | Longyan3 | 0 | 0 | 0 | 0 | 0 | 0 | 138 | 0 | 0 | 0 | 0 | 0 | 0 | 0 | 0 | 0 |  |
|  | Longyan4 | 0 | 1 | 2 | 0 | 0 | 0 | 0 | 104 | 14 | 0 | 0 | 1 | 1 | 0 | 4 | 6 |  |
|  | Brave1 | 7 | 4 | 1 | 2 | 0 | 2 | 0 | 15 | 113 | 0 | 2 | 0 | 0 | 0 | 0 | 2 |  |
|  | Morgan | 0 | 0 | 0 | 0 | 0 | 1 | 0 | 0 | 0 | 135 | 0 | 0 | 0 | 0 | 0 | 0 |  |
|  | Monica | 0 | 0 | 9 | 34 | 0 | 0 | 0 | 6 | 1 | 0 | 129 | 4 | 0 | 0 | 0 | 2 |  |
|  | Tanke | 0 | 0 | 1 | 2 | 0 | 0 | 0 | 1 | 0 | 0 | 1 | 130 | 1 | 0 | 0 | 0 |  |
|  | Youmu1 | 0 | 4 | 11 | 1 | 0 | 0 | 0 | 0 | 1 | 0 | 0 | 1 | 122 | 0 | 1 | 0 |  |
|  | Baiyan7 | 1 | 0 | 0 | 0 | 0 | 4 | 0 | 0 | 0 | 0 | 0 | 0 | 0 | 121 | 1 | 0 |  |
|  | Dingyan2 | 2 | 1 | 1 | 0 | 0 | 0 | 0 | 2 | 0 | 0 | 0 | 0 | 0 | 1 | 99 | 14 |  |
|  | Quebec | 0 | 0 | 4 | 0 | 0 | 0 | 0 | 2 | 0 | 0 | 0 | 0 | 1 | 0 | 20 | 101 |  |
|  | **Accuracy (%)** | 83.57 | 89.29 | 66.43 | 69.29 | 87.86 | 92.14 | 98.57 | 74.29 | 80.71 | 96.43 | 92.14 | 92.86 | 87.14 | 86.43 | 70.71 | 72.14 | 83.88 |
| Testing | Blade | 50 | 1 | 1 | 0 | 0 | 0 | 0 | 0 | 0 | 0 | 0 | 0 | 0 | 2 | 0 | 0 |  |
| (60) | Deon | 4 | 53 | 6 | 0 | 0 | 0 | 1 | 1 | 4 | 0 | 0 | 0 | 1 | 1 | 4 | 4 |  |
|  | Jerry | 0 | 4 | 40 | 2 | 0 | 0 | 0 | 1 | 0 | 0 | 0 | 2 | 4 | 0 | 0 | 2 |  |
|  | Kona | 0 | 0 | 1 | 43 | 0 | 0 | 0 | 2 | 0 | 0 | 3 | 0 | 0 | 0 | 3 | 0 |  |
|  | Longyan1 | 0 | 0 | 0 | 0 | 54 | 3 | 0 | 0 | 0 | 1 | 0 | 0 | 0 | 0 | 0 | 0 |  |
|  | Longyan2 | 2 | 0 | 0 | 0 | 6 | 52 | 0 | 0 | 1 | 0 | 0 | 0 | 0 | 5 | 0 | 0 |  |
|  | Longyan3 | 0 | 0 | 0 | 0 | 0 | 0 | 59 | 0 | 0 | 0 | 0 | 0 | 0 | 0 | 0 | 0 |  |
|  | Longyan4 | 0 | 0 | 1 | 1 | 0 | 0 | 0 | 39 | 5 | 0 | 0 | 1 | 1 | 0 | 1 | 5 |  |
|  | Brave1 | 2 | 2 | 1 | 1 | 0 | 1 | 0 | 8 | 47 | 0 | 1 | 0 | 0 | 0 | 0 | 1 |  |
|  | Morgan | 0 | 0 | 0 | 0 | 0 | 1 | 0 | 0 | 0 | 59 | 0 | 0 | 0 | 0 | 0 | 0 |  |
|  | Monica | 0 | 0 | 5 | 13 | 0 | 0 | 0 | 4 | 2 | 0 | 55 | 0 | 0 | 0 | 0 | 0 |  |
|  | Tanke | 0 | 0 | 0 | 0 | 0 | 0 | 0 | 1 | 0 | 0 | 1 | 57 | 0 | 0 | 0 | 0 |  |
|  | Youmu1 | 0 | 0 | 4 | 0 | 0 | 0 | 0 | 0 | 0 | 0 | 0 | 0 | 53 | 0 | 0 | 0 |  |
|  | Baiyan7 | 1 | 0 | 0 | 0 | 0 | 3 | 0 | 0 | 0 | 0 | 0 | 0 | 0 | 52 | 0 | 0 |  |
|  | Dingyan2 | 1 | 0 | 0 | 0 | 0 | 0 | 0 | 2 | 1 | 0 | 0 | 0 | 0 | 0 | 45 | 10 |  |
|  | Quebec | 0 | 0 | 1 | 0 | 0 | 0 | 0 | 2 | 0 | 0 | 0 | 0 | 1 | 0 | 7 | 38 |  |
|  | **Accuracy (%)** | 83.33 | 88.33 | 66.67 | 71.67 | 90.00 | 86.67 | 98.33 | 65.00 | 78.33 | 98.33 | 91.67 | 95.00 | 88.33 | 86.67 | 75.00 | 63.33 | 80.92 |
