# Supplementary material for: Cultivars identification of oat (Avena sativa L.) seed via multispectral imaging analysis
Source: Front Plant Sci. 2023 Feb 7;14:1113535. doi: 10.3389/fpls.2023.1113535 (PMC9941542; doi:10.3389/fpls.2023.1113535)
Supplement: Supplementary file 4 [file Table_4.docx]

Supplementary Table 4. Discrimination performance based on SVM with morphology features of 16 *Avena sativa* L. cultivars.

|  | **Predict** | **Actual** | | | | | | | | | | | | | | | | **Total (%)** |
| --- | --- | --- | --- | --- | --- | --- | --- | --- | --- | --- | --- | --- | --- | --- | --- | --- | --- | --- |
|  |  | Blade | Deon | Jerry | Kona | Longyan1 | Longyan2 | Longyan3 | Longyan4 | Brave1 | Morgan | Monica | Tanke | Youmu1 | Baiyan7 | Dingyan2 | Quebec |  |
| Training | Blade | 115 | 3 | 2 | 0 | 0 | 0 | 0 | 0 | 0 | 0 | 0 | 0 | 7 | 10 | 0 | 1 |  |
| (140) | Deon | 5 | 83 | 8 | 6 | 0 | 0 | 0 | 0 | 6 | 1 | 0 | 0 | 17 | 7 | 3 | 4 |  |
|  | Jerry | 7 | 12 | 94 | 13 | 2 | 0 | 0 | 0 | 6 | 1 | 0 | 1 | 6 | 5 | 0 | 1 |  |
|  | Kona | 0 | 2 | 15 | 66 | 0 | 20 | 0 | 10 | 17 | 0 | 15 | 1 | 0 | 1 | 9 | 2 |  |
|  | Longyan1 | 0 | 0 | 1 | 2 | 107 | 12 | 0 | 2 | 2 | 0 | 6 | 2 | 0 | 4 | 0 | 2 |  |
|  | Longyan2 | 1 | 0 | 1 | 16 | 14 | 71 | 0 | 8 | 17 | 0 | 12 | 3 | 0 | 9 | 10 | 14 |  |
|  | Longyan3 | 0 | 0 | 0 | 0 | 0 | 0 | 139 | 0 | 0 | 0 | 0 | 0 | 0 | 0 | 0 | 0 |  |
|  | Longyan4 | 0 | 0 | 0 | 3 | 3 | 10 | 0 | 102 | 8 | 0 | 4 | 2 | 0 | 2 | 3 | 9 |  |
|  | Brave1 | 0 | 3 | 9 | 3 | 3 | 7 | 0 | 4 | 61 | 0 | 3 | 2 | 1 | 3 | 3 | 10 |  |
|  | Morgan | 0 | 1 | 0 | 0 | 0 | 0 | 0 | 0 | 0 | 131 | 0 | 0 | 5 | 0 | 0 | 0 |  |
|  | Monica | 0 | 0 | 4 | 24 | 8 | 6 | 0 | 3 | 10 | 0 | 100 | 1 | 0 | 0 | 0 | 1 |  |
|  | Tanke | 1 | 0 | 2 | 1 | 2 | 2 | 0 | 3 | 2 | 0 | 0 | 126 | 1 | 0 | 0 | 0 |  |
|  | Youmu1 | 2 | 24 | 2 | 0 | 0 | 1 | 0 | 0 | 1 | 7 | 0 | 2 | 86 | 6 | 2 | 0 |  |
|  | Baiyan7 | 5 | 7 | 2 | 0 | 0 | 1 | 0 | 1 | 2 | 0 | 0 | 0 | 11 | 69 | 3 | 27 |  |
|  | Dingyan2 | 2 | 4 | 0 | 5 | 1 | 3 | 1 | 3 | 3 | 0 | 0 | 0 | 6 | 10 | 98 | 20 |  |
|  | Quebec | 2 | 1 | 0 | 1 | 0 | 7 | 0 | 4 | 5 | 0 | 0 | 0 | 0 | 14 | 9 | 49 |  |
|  | **Accuracy (%)** | 82.14 | 59.29 | 67.14 | 47.14 | 76.43 | 50.71 | 99.29 | 72.86 | 43.57 | 93.57 | 71.43 | 90.00 | 61.43 | 49.29 | 70.00 | 35.00 | 66.83 |
| Testing | Blade | 46 | 0 | 1 | 0 | 0 | 0 | 0 | 0 | 0 | 0 | 0 | 0 | 6 | 3 | 0 | 0 |  |
| (60) | Deon | 4 | 33 | 0 | 1 | 0 | 0 | 0 | 0 | 7 | 1 | 0 | 0 | 5 | 3 | 1 | 4 |  |
|  | Jerry | 4 | 6 | 41 | 2 | 0 | 0 | 0 | 0 | 3 | 1 | 0 | 0 | 1 | 1 | 0 | 1 |  |
|  | Kona | 0 | 0 | 7 | 26 | 0 | 6 | 0 | 5 | 10 | 0 | 9 | 1 | 0 | 0 | 1 | 1 |  |
|  | Longyan1 | 0 | 1 | 1 | 1 | 47 | 8 | 0 | 1 | 1 | 1 | 4 | 1 | 0 | 3 | 0 | 1 |  |
|  | Longyan2 | 0 | 0 | 0 | 8 | 6 | 32 | 0 | 6 | 4 | 0 | 3 | 3 | 0 | 2 | 4 | 7 |  |
|  | Longyan3 | 0 | 0 | 0 | 0 | 0 | 0 | 60 | 0 | 0 | 0 | 0 | 0 | 0 | 0 | 0 | 0 |  |
|  | Longyan4 | 0 | 0 | 0 | 1 | 2 | 5 | 0 | 41 | 4 | 0 | 2 | 0 | 0 | 1 | 1 | 2 |  |
|  | Brave1 | 1 | 2 | 4 | 2 | 0 | 1 | 0 | 3 | 18 | 0 | 1 | 0 | 1 | 1 | 1 | 5 |  |
|  | Morgan | 0 | 0 | 0 | 0 | 0 | 0 | 0 | 0 | 0 | 55 | 0 | 0 | 3 | 0 | 0 | 0 |  |
|  | Monica | 0 | 0 | 1 | 18 | 3 | 2 | 0 | 0 | 5 | 0 | 41 | 0 | 0 | 0 | 0 | 0 |  |
|  | Tanke | 1 | 0 | 3 | 0 | 1 | 0 | 0 | 1 | 2 | 0 | 0 | 55 | 0 | 0 | 0 | 0 |  |
|  | Youmu1 | 3 | 9 | 2 | 0 | 0 | 0 | 0 | 0 | 0 | 2 | 0 | 0 | 38 | 1 | 0 | 0 |  |
|  | Baiyan7 | 1 | 5 | 0 | 0 | 0 | 0 | 0 | 0 | 1 | 0 | 0 | 0 | 5 | 36 | 0 | 11 |  |
|  | Dingyan2 | 0 | 4 | 0 | 1 | 1 | 2 | 0 | 3 | 2 | 0 | 0 | 0 | 1 | 5 | 46 | 7 |  |
|  | Quebec | 0 | 0 | 0 | 0 | 0 | 4 | 0 | 0 | 3 | 0 | 0 | 0 | 0 | 4 | 6 | 21 |  |
|  | **Accuracy (%)** | 76.67 | 55.00 | 68.33 | 43.33 | 78.33 | 53.33 | 100.00 | 68.33 | 30.00 | 91.66 | 68.33 | 91.66 | 63.33 | 60.00 | 76.67 | 35.00 | 66.25 |
