# Supplementary material for: Cultivars identification of oat (Avena sativa L.) seed via multispectral imaging analysis
Source: Front Plant Sci. 2023 Feb 7;14:1113535. doi: 10.3389/fpls.2023.1113535 (PMC9941542; doi:10.3389/fpls.2023.1113535)
Supplement: Supplementary file 5 [file Table_5.docx]

Supplementary Table 5. Discrimination performance based on SVM with spectral features of 16 *Avena sativa* L. cultivars.

|  | **Predict** | **Actual** | | | | | | | | | | | | | | | | **Total (%)** |
| --- | --- | --- | --- | --- | --- | --- | --- | --- | --- | --- | --- | --- | --- | --- | --- | --- | --- | --- |
|  |  | Blade | Deon | Jerry | Kona | Longyan1 | Longyan2 | Longyan3 | Longyan4 | Brave1 | Morgan | Monica | Tanke | Youmu1 | Baiyan7 | Dingyan2 | Quebec |  |
| Training | Blade | 132 | 0 | 0 | 0 | 0 | 0 | 0 | 0 | 1 | 0 | 0 | 0 | 0 | 0 | 1 | 0 |  |
| (140) | Deon | 5 | 127 | 12 | 0 | 0 | 0 | 0 | 0 | 4 | 0 | 0 | 0 | 7 | 2 | 6 | 6 |  |
|  | Jerry | 0 | 4 | 103 | 2 | 0 | 0 | 0 | 4 | 2 | 0 | 0 | 1 | 13 | 1 | 4 | 7 |  |
|  | Kona | 0 | 0 | 2 | 122 | 0 | 0 | 0 | 0 | 0 | 0 | 5 | 4 | 0 | 0 | 2 | 0 |  |
|  | Longyan1 | 0 | 0 | 0 | 0 | 138 | 4 | 0 | 0 | 0 | 1 | 0 | 0 | 0 | 1 | 0 | 0 |  |
|  | Longyan2 | 1 | 0 | 0 | 0 | 1 | 134 | 0 | 0 | 0 | 0 | 0 | 0 | 0 | 7 | 0 | 0 |  |
|  | Longyan3 | 0 | 0 | 0 | 0 | 0 | 0 | 140 | 0 | 0 | 0 | 0 | 0 | 0 | 0 | 0 | 0 |  |
|  | Longyan4 | 0 | 0 | 2 | 0 | 0 | 0 | 0 | 123 | 9 | 0 | 3 | 0 | 0 | 0 | 2 | 4 |  |
|  | Brave1 | 0 | 3 | 2 | 0 | 0 | 1 | 0 | 3 | 115 | 1 | 1 | 0 | 0 | 1 | 2 | 4 |  |
|  | Morgan | 0 | 0 | 0 | 0 | 0 | 0 | 0 | 0 | 0 | 138 | 0 | 0 | 0 | 0 | 0 | 0 |  |
|  | Monica | 0 | 0 | 0 | 10 | 0 | 0 | 0 | 2 | 0 | 0 | 129 | 2 | 0 | 0 | 0 | 0 |  |
|  | Tanke | 0 | 0 | 0 | 3 | 0 | 0 | 0 | 0 | 0 | 0 | 2 | 132 | 0 | 0 | 0 | 0 |  |
|  | Youmu1 | 0 | 2 | 9 | 0 | 0 | 0 | 0 | 0 | 0 | 0 | 0 | 1 | 118 | 0 | 0 | 0 |  |
|  | Baiyan7 | 1 | 1 | 0 | 0 | 1 | 1 | 0 | 0 | 0 | 0 | 0 | 0 | 0 | 126 | 0 | 1 |  |
|  | Dingyan2 | 1 | 3 | 7 | 1 | 0 | 0 | 0 | 2 | 5 | 0 | 0 | 0 | 1 | 1 | 107 | 13 |  |
|  | Quebec | 0 | 0 | 3 | 2 | 0 | 0 | 0 | 6 | 4 | 0 | 0 | 0 | 1 | 1 | 16 | 105 |  |
|  | **Accuracy (%)** | 94.29 | 90.71 | 73.57 | 87.14 | 98.57 | 95.71 | 100.00 | 87.86 | 82.14 | 98.57 | 92.14 | 94.29 | 84.29 | 90.00 | 76.43 | 75.00 | 88.79 |
| Testing | Blade | 55 | 0 | 0 | 0 | 0 | 0 | 0 | 0 | 1 | 0 | 0 | 0 | 0 | 0 | 0 | 0 |  |
| (60) | Deon | 2 | 57 | 5 | 0 | 0 | 0 | 0 | 0 | 3 | 0 | 0 | 0 | 1 | 1 | 4 | 4 |  |
|  | Jerry | 0 | 3 | 46 | 0 | 0 | 0 | 0 | 2 | 1 | 0 | 0 | 0 | 7 | 0 | 1 | 0 |  |
|  | Kona | 0 | 0 | 0 | 54 | 0 | 0 | 0 | 0 | 0 | 0 | 1 | 0 | 0 | 0 | 0 | 0 |  |
|  | Longyan1 | 0 | 0 | 0 | 0 | 57 | 1 | 0 | 0 | 0 | 1 | 0 | 0 | 0 | 0 | 0 | 0 |  |
|  | Longyan2 | 1 | 0 | 0 | 0 | 3 | 57 | 0 | 0 | 0 | 0 | 0 | 0 | 0 | 3 | 0 | 0 |  |
|  | Longyan3 | 0 | 0 | 0 | 0 | 0 | 0 | 60 | 0 | 0 | 0 | 0 | 0 | 0 | 0 | 0 | 0 |  |
|  | Longyan4 | 0 | 0 | 0 | 0 | 0 | 0 | 0 | 48 | 4 | 0 | 0 | 0 | 0 | 0 | 1 | 3 |  |
|  | Brave1 | 1 | 0 | 1 | 0 | 0 | 0 | 0 | 2 | 47 | 0 | 0 | 0 | 0 | 1 | 0 | 1 |  |
|  | Morgan | 0 | 0 | 0 | 0 | 0 | 0 | 0 | 0 | 0 | 58 | 0 | 0 | 0 | 0 | 0 | 0 |  |
|  | Monica | 0 | 0 | 0 | 3 | 0 | 0 | 0 | 1 | 0 | 0 | 59 | 1 | 0 | 0 | 0 | 0 |  |
|  | Tanke | 0 | 0 | 0 | 2 | 0 | 0 | 0 | 0 | 0 | 0 | 0 | 59 | 0 | 0 | 0 | 0 |  |
|  | Youmu1 | 0 | 0 | 5 | 0 | 0 | 0 | 0 | 1 | 0 | 0 | 0 | 0 | 51 | 0 | 0 | 0 |  |
|  | Baiyan7 | 0 | 0 | 1 | 0 | 0 | 2 | 0 | 0 | 0 | 1 | 0 | 0 | 0 | 53 | 0 | 0 |  |
|  | Dingyan2 | 1 | 0 | 1 | 1 | 0 | 0 | 0 | 1 | 3 | 0 | 0 | 0 | 0 | 0 | 50 | 12 |  |
|  | Quebec | 0 | 0 | 1 | 0 | 0 | 0 | 0 | 5 | 1 | 0 | 0 | 0 | 1 | 2 | 4 | 40 |  |
|  | **Accuracy (%)** | 91.67 | 95.00 | 76.67 | 90.00 | 95.00 | 95.00 | 100.00 | 80.00 | 78.33 | 96.67 | 98.33 | 98.33 | 85.00 | 88.33 | 83.33 | 66.67 | 88.65 |
